# *Streptococcus Pneumoniae*-associated Thrombotic Microangiopathy in an Immunosuppressed Adult

**DOI:** 10.1515/med-2020-0030

**Published:** 2020-03-19

**Authors:** Yumi Ichikawa, Masato Murata, Makoto Aoki, Jun Nakajima, Yuta Isshiki, Yusuke Sawada, Kazunori Fukushima, Kiyohiro Oshima

**Affiliations:** 1 Department of Emergency Medicine, Gunma University Graduate School of Medicine, 3-19-15 Showamachi, Maebashi, 371-8511, Gunma, Japan; 2 Emergency Medical Center, Gunma University Hospital, 3-19-15 Showa-machi, Maebashi, 371-8511, Gunma, Japan

**Keywords:** Hemolytic uremic syndrome, Antibiotics, Plasma exchange, Infection

## Abstract

A 62-year-old male who was receiving prednisolone and methotrexate for scleroderma and rheumatoid arthritis complained of diarrhea and vomiting, and was transferred to our hospital for detailed examination and treatment of renal dysfunction and thrombocytopenia. Hemolytic anemia and crushed erythrocytes were found during the patient’s course; therefore, we suspected thrombotic microangiopathy (TMA). His ADAMTS13 activity was 60.3% and his ADAMTS13 inhibitor was under 0.5. In addition, his blood culture was positive for *Streptococcus pneumoniae*, and we finally diagnosed *Streptococcus pneumoniae*-associated TMA (pTMA). The patient was treated with antibiotics and hemodialysis. The patient recovered and was discharged on the 45^th^ hospital day. Adult pTMA cases are remarkably rare. We herein report a successfully treated adult case of pTMA.

## 1 Introduction

Thrombotic microangiopathy (TMA) is defined as microangiopathic anemia, thrombocytopenia and microvascular ischemia affecting various organs. Among *Streptococcus pneumoniae (S. pneumoniae)* diseases, invasive pneumococcal diseases cause *S. pneumoniae*-associated TMA (pTMA). Most cases of pTMA occur in neonates and children aged less than 2 years old, and adult cases are remarkably rare [1]. We herein report a case of pTMA in an adult patient with a literature review.

## 2 Case

A 62-year-old male with scleroderma, rheumatoid arthritis, diabetes mellitus and hypertension was taking 5mg/day of prednisolone and 8mg/week of methotrexate. The patient had originally been prescribed 20mg/day of prednisolone and the dosage was tapered to 5 mg/day 11 months previously. The patient had started to take methotrexate 10 days previously. Then, the patient complained of diarrhea, vomiting and chills. He was admitted to the previous hospital with the diagnosis of infectious enteritis and was treated with ceftriaxone. Two days later, he was transferred to the emergency room of our hospital for investigation and treatment of renal dysfunction and thrombocytopenia.

**Figure 1:**
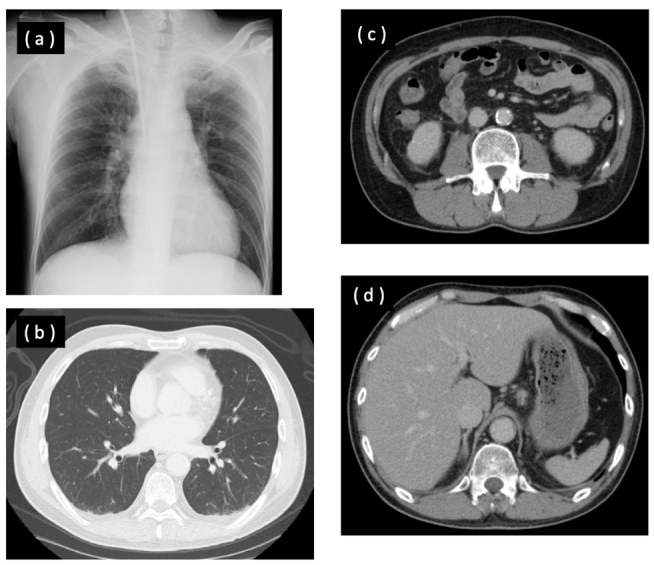
(a) Chest roentgenogram on arrival at our hospital showed no abnormal findings. (b) Chest computed tomography (CT) on arrival showed no abnormal findings. (c) Abdominal CT on arrival showed no abnormal findings. (d) Abdominal CT on arrival showed neither splenomegaly nor atrophy of the spleen.

In our emergency room, the patient was alert. His body temperature was 36.9°C, heart rate 89/min, blood pressure 148/85 mmHg, SpO_2_ 99 % (ambient air), and respiratory rate 9/min. He had facial edema. Laboratory evaluation on admission revealed thrombocytopenia, elevated white blood cell count, elevated C-reactive protein level, abnormality of the coagulation-fibrinolytic system, and hepatorenal dysfunction (Table 1). Chest roentgenogram and thoracoabdominal contrast-enhanced computed tomography (CT) revealed no evidence of infection (Fig. 1a-c). There was no splenomegaly nor atrophy of the spleen on CT (Fig. 1d).

**Table 1:** Laboratory Data

Laboratory Data on Admission					
Peripheral Blood			Blood Chemistry		
WBC	16,7	/μL	CRP	25.62	mg/dL
(Neu 95.5%, Eos 0.0%, Bas 0.1%, Mo 0.9%, Lym 3.5%)			TP	5-Aug	g/dL
Hb	13.0	g/dL	Alb	3.0	g/dL
Plt	2-Apr	×10^4^/μL	AST	329	IU/L
			ALT	156	IU/L
Blood Coagulation			ALP	412	IU/L
FDP	800	μg/mL	LDH	1678	IU/L
D-dimer	344.1	μg/mL	γ-GTP	92	IU/L
PT ratio	Jan-59		T-Bil	2-Sep	mg/dL
Fibrinogen	145	mg/dL	BUN	50	mg/dL
			Cr	Mar-73	mg/dL
			Na	136	mEq/L
			K	5-Mar	mEq/L
			Cl	98	mEq/L
			BS	189	mg/dl

Laboratory Data on Day 2			Laboratory Data on Day 4		
CH-50	45.6	U/min	T-Bil	1-Jun	mg/dL
			D-Bil	0.2	mg/dL
			Haptoglobin	<6.6	mg/dL

Alb, Serum albumin; ALP, Alkaline phosphatase; ALT, Alanine aminotransferase; AST, Aspartate aminotransferase; Bas, Basophils; BS, Fasting blood glucose; BUN, Blood urea nitrogen; CH-50, 50% hemolytic complement activity; Cl, Serum chloride; Cr, Serum creatinine; CRP, C-reactive protein; D-Bil, Serum direct bilirubin; Eos, Eosinophils; FDP, Fibrin degradation product; Hb, Hemoglobin; K, Serum potassium; LDH, Lactate dehydrogenase; Lym, Lymphocytes; Mo, Monocytes; Na, Serum sodium; Neu, Neutrophils; Plt, Platelet count; PT, Prothrombin time; T-Bil, Serum total bilirubin; TP, Total protein; WBC, White blood cell count; γ-GTP, γ-glutamyl transpeptidase

**Figure 2:**
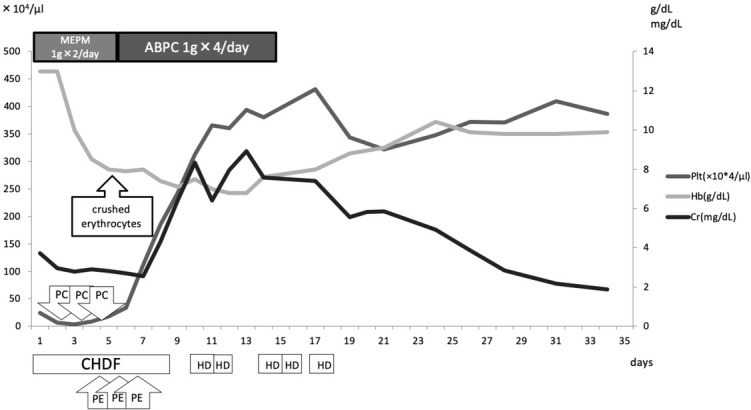
Our patient’s clinical course. Crushed erythrocytes were found in the peripheral blood smear performed at the previons hospital, on the 5th hospital day. ABPC: ampicillin; CHDF: continuous hemodiafiltration; Cr: creatinine; Hb: hemoglobin; HD: hemodialysis; MEPM: meropenem; PC: platelet concentrate; Plt: platelet count; PE: plasma exchange.

**Figure 3:**
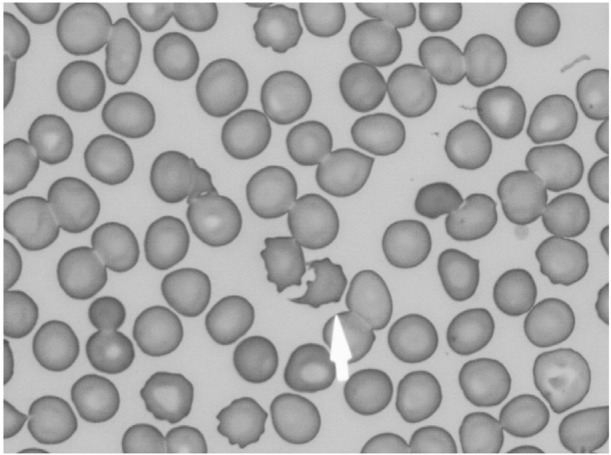
Crushed erythrocytes (arrow) in a peripheral blood smear stained with May-Giemsa stain (x1000).

We considered that he had disseminated intravascular coagulation (DIC) based on an infectious disease, and he was moved to the intensive care unit (ICU). His clinical course is shown in Fig. 2. Continuous hemodiafiltration (CHDF) was started for renal dysfunction on the 1^st^ hospital day. On the 3rd hospital day, his hemoglobin level and platelet count decreased to 10g/dl and 3,000/μl, respectively (Fig. 2), and crushed erythrocytes were found in the peripheral blood smear performed on the 5th hospital day (Fig. 3). Differential diagnoses included TMA, hemolytic uremic syndrome (HUS) and thrombotic thrombocytopenic purpura (TTP) based on his thrombocytopenia, crushed erythrocytes and renal dysfunction, and plasma exchange (PE) was performed. TTP was ruled out among the differential diagnoses because his ADAMTS13 activity was 60.3% and his ADAMTS 13 inhibitor was under 0.5. On the 5th hospital day, *S. pneumoniae* was detected in his blood culture performed at the previous hospital; therefore, we made the final diagnosis of pTMA. We stopped PE and changed the antibiotic from meropenem to ampicillin. The antibiogram of the *Streptococcus pneumoniae* performed at the previous hospital is shown in Table 2. The *Streptococcus pneumoniae* did not have penicillin resistance. We initially considered treating the patient with benzylpenicillin potassium (PCG). However, because the patient had renal damage and hyperkalemia, we chose to treat the patient with ampicillin.

**Table 2:** Antibiogram of the *Streptococcus pneumoniae* isolated from our patient

Antibiotic	MIC_50_(μg/mL)	Susceptibility
		(S, susceptible; R, resistant)
PCG	0.06	S
AMPC	-	S
CVA/AMPC	-	S
CTX	≤0.06	S
CTM	≤0.25	S
CFPN-PI	-	S
CTDR-PI	-	S
PAPM/BP	≤0.12	S
MEPM	≤0.12	S
EM	≥2	R
CAM	-	R
CLDM	-	S
TC	-	R
VCM	≤0.25	S
TFLX	-	S
LVFX	-	S

AMPC, amoxicillin; CAM, clarithromycin; CFPN-PI, cefcapene pivoxil; CLDM, clindamycin; CTDR-PI, cefditoren pivoxil; CTM, cefotiam; CTX, cefotaxime; CVA/AMPZ, clavulanate/amoxicillin; EM, erythromycin; LVFX, levofloxacin; MEPM, meropenem; MIC, minimum inhibitory concentration; TC, tetracycline; TFLX, tosufloxacin; PAPM/BP, panipenem/ betamipron, PCG, benzylpenicillin potassium; VCM, vancomycin

The patient’s hemodynamics became stable and CHDF was switched to hemodialysis (HD) on the 8th hospital day. His condition gradually improved. He was transferred from the ICU to the normal ward on the 13th hospital day, and HD was discontinued on the 17th hospital day. He was discharged from our hospital on the 45th hospital day.

Informed consent has been obtained from patient included in this study.

## 3 Discussion

**Figure 4:**
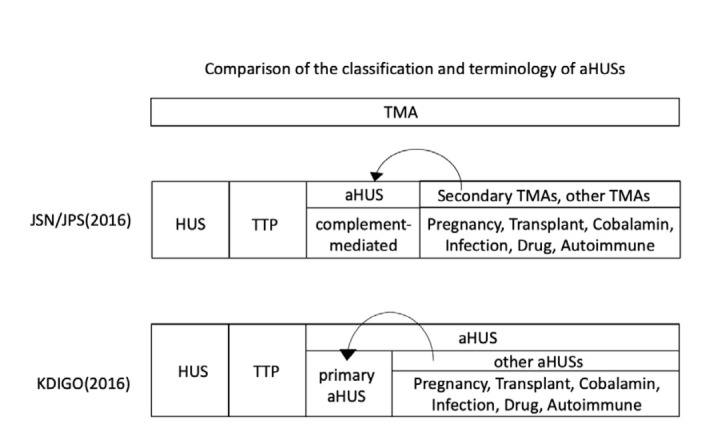
Comparison of the classification of TMAs. In the classification of JSN/JPS, a genetic or complement abnormality can be found and the differentiation between atypical hemolytic uremic syndrome (aHUS) and secondary TMAs is sometimes difficult. Patients with true aHUS (i.e., complement-mediated aHUS) are sometimes diagnosed as having secondary TMA. In the classification of KDIGO, the differentiation between primary aHUS and other aHUSs, such as pregnancy, transplant are sometimes difficult. Patients with primary aHUS are sometimes diagnosed as having another type of aHUS [4]. JSN/JPS, Japanese Society of Nephrology and the Japan Pediatric Society; TMA, Thrombotic microangiopathy; aHUS, atypical hemolytic uremic syndrome; HUS, hemolytic uremic syndrome; TTP, thrombotic thrombocytopenic purpura; KDIGO, Kidney Disease Improving Global Outcomes.

The Japanese Clinical Guide of Atypical Hemolytic Uremic Syndrome (aHUS) published by the Japanese Society of Nephrology and the Japan Pediatric Society (JSN/JPS) [2] and the “Kidney Disease: Improving Global Outcomes (KDIGO)” Controversies Conference reported a consensus of classification of TMAs [3] (Fig. 4). In the Japanese Clinical Guide, aHUS was defined as complement-mediated HUS after the exclusion of Shiga toxin-producing *Escherichia coli* (STEC)-HUS, TTP and secondary TMAs [2]. At the KDIGO Controversies Conference, TMA and all secondary TMAs were again classified under aHUS; however, even if TMAs are categorized as aHUS, it does not necessarily mean that all of these diseases are congenital or acquired complement-mediated HUS [3]. pTMA is secondary to TMA caused by infection. Sometimes differential diagnosis of TMA among secondary TMAs including HELLP syndrome is difficult, and complement-mediated HUS patients have been reported [4].

In our patient, we finally obtained the diagnosis of TMA because he had microangiopathic anemia, thrombocytopenia and microvascular ischemia affecting various organs. We initially suspected that the crushed erythrocytes were produced by plasma exchange and CHDF, but this was ruled out because crushed erythrocytes were found in the specimen obtained at the previous hospital. We assessed that he did not suffer from TTP because his ADAMTS13 activity was normal and ADAMTS13 inhibitor was not increased. *S. pneumoniae* was detected in the blood culture at the previous hospital; therefore, we concluded that he suffered from pTMA. The patient had begun living together with his 4-year-old grandchild since one month before the onset of pTMA. Although the grandchild had received pneumococcal vaccination, the grandchild may have been the infection route.

Pneumococcal infectious diseases, particularly invasive pneumococcal diseases, can cause pTMA. Invasive pneumococcal disease is defined as severe pneumococcal disease causing meningitis, bacteremia, sepsis or thoracic empyema. Although pneumococcal disease is common in both children and adults, pTMA usually occurs in children [5,6]. There is a possibility that splenectomy and immunosuppression are associated with pTMA in adults [6]. Our patient was immunosuppressed with prednisolone and methotrexate, and there is a possibility that immunosuppression caused pTMA.

In our case, antibiotics and temporary hemodialysis improved his renal function. Infection can cause complement-related aHUS, and mutation of a gene encoding a regulatory protein in the complement system can be related to the prognosis of patients with complement-related aHUS [7]. However, in our case, he had no mutation of the genes encoding proteins in the complement system. On the other hand, all of the gene mutations and complement abnormality of complement-related aHUS are not clear [7], and it is possible that genetic mutation of an unknown gene or abnormality of complement participated in the etiology and prognosis of the present case.

Endothelial injury activates a microangiopathic cascade of thrombotic vascular injury and leads to TMA [5]. In pTMA, *S. pneumoniae* cleaves N-acetylneuraminic acid and exposes the Thomsen–Friedenreich antigen (T-antigen) on glomerular endothelial cell glycoproteins [8]. This process leads to IgM binding from circulating anti-T IgM antibodies, and the clinical syndrome of TMA [9].

Medical treatment of pTMA consists of administration of antibiotics against *Streptococcus pneumoniae* and symptomatic care. If necessary, transfusion of washed blood products is preferable to avoid increasing the levels of preformed anti-T antibodies, whose levels are high in unwashed products [1,9]. PE with 5% albumin replacement fluid has been advocated for the treatment of pTMA due to the theoretical benefit of removing anti-T IgM antibodies and bacterial neuraminidase. On the other hand, previous reports indicated that PE with fresh frozen plasma was not recommended for the treatment of pTMA because preformed anti-T antibodies in the pooled product were said to worsen the condition of patients with pTMA [1,9]. In addition, the effect of PE in adult pTMA cases is uncertain. Maki et al. [6] reviewed five adult pTMA cases in the literature [6, 10-13]. We summarize the clinical features of the 10 reported cases and the present case in Table 3. PE was performed in 8 patients and HD was performed in 7 patients. Renal function recovered in 9 cases including the present case. One patient finally underwent renal transplantation even though PE and HD were performed. Another patient finally died even though antibiotic therapy and PE were performed. Accumulation of additional cases is necessary to clarify the effect of PE in adult pTMA patients.

**Table 3:** Cases of pTMA in adult patients

Authors	Age (yr)	Sex	Underlying factor	SP infection	Antibiotics	PE	HD	Renal outcome
von Eyben et al.[10]	35	M	Post-splenectomy	Sepsis	PC	-	+	Improved
Myers et al.[11]	50	F	None	Sepsis	ABPC+GM	+	+	Renal transplantation
Ohlmann et al.[12]	52	F	None	Sepsis	CTRX	+	+	Improved
Reynolds et al.[13]	41	F	None	Pneumonia	CTRX	+	+	Improved
Maki et al.[6]	62	F	Post-splenectomy	Upper tract infection respiratory	MINO	-	-	Improved
Allen et al.[14]	83	F	None	Sepsis	CAM +CVA/AMPC	+	+	Improved
Saraceni et al.[15]	46	F	Post-splenectomy	Sepsis	CTRX	-	-	Improved
Yang et al.[16]	50	M	None	Sepsis	CTRX+VCM	+	-	Improved
Ishiguro et al.[17]	65	M	chronic pulmonary obstructive disease	Sepsis	CFPM+AZM	+	-	Died
Jeante et al.[18]	53	M	type and alcohol 2 diabetes abuse mellitus	Pneumonia	CTRX+LVFX	+	+	Improved
Present case	62	M	oral immunosuppressant	Sepsis	MEPM+ABPC	+	+	Improved

We experienced a successfully treated adult pTMA case. The occurrence of pTMA is rare; therefore, further cases are necessary to clarify useful treatments for adult pTMA patients.

## Abbreviations

aHUS: hemolytic uremic syndrome
CHDF: continuous hemodiafiltration
DIC: disseminated intravascular coagulation
HD: hemodialysis
HUS: hemolytic uremic syndrome
JSN/JPS: Japanese Society of Nephrology and the Japan Pediatric Society
KDIGO: Kidney Disease: Improving Global Outcomes
PE: plasma exchange
pTMA: *Streptococcus pneumoniae*-associated thrombotic microangiopathy
STEC: Shiga toxin-producing *Escherichia coli*
TMA: thrombotic microangiopathy
TTP: thrombotic thrombocytopenic purpura
